# Stricture of the Urethra

**Published:** 1894-03

**Authors:** 


Stricture of the Urethra. By G. Frank Lydston, M. D., Chicago. The W. T. Keener Company, 1893.

An excellent book for the student, being clear and concise and more conservative in the treatment advocated, than many works by specialists. While Dr. Lydston gives the various methods of treatment in vogue at the present time, he never fails to express his convictions as to the treatment which he considers most advisable. The illustrations are largely reproductions of well known originals. There are an unnecessarily large number of typographical errors, which detract somewhat from the pleasure of the book.

